# *Pseudomonas aeruginosa* device associated *–* healthcare associated infections and its multidrug resistance at intensive care unit of University Hospital: polish, 8.5-year, prospective, single-centre study

**DOI:** 10.1186/s12879-021-05883-5

**Published:** 2021-02-16

**Authors:** Agnieszka Litwin, Stanislaw Rojek, Waldemar Gozdzik, Wieslawa Duszynska

**Affiliations:** 1 Microbiology Laboratory, University Hospital Wroclaw, Borowska Street 213, Wroclaw, Poland; 2 Department and Clinic of Anaesthesiology and Intensive Therapy, Wroclaw Medical University, L. Pasteura Street 1, 50-367 Wroclaw, Poland

**Keywords:** *Pseudomonas aeruginosa*, DA-HAIs, Multidrug resistance

## Abstract

**Background:**

*Pseudomonas aeruginosa* has recently shown to be one of the most important strains of bacteria and alert pathogens in Europe among Intensive Care Unit patients that provide serious therapeutic problems because of its multidrug resistance.

**Methods:**

The purpose of this microbiological study was data analysis of device associated- healthcare associated infections (DA-HAIs) in an ICU in terms of the incidents of *P.aeruginosa* strain infections and its susceptibility within an 8.5-year observation.

**Results:**

Among 919 isolated strains responsible for 799 DA-HAIs (17,62 ± 1,98/1000 patient-days) in 4010 ICU patients *P.aeruginosa* was the pathogen in 108/799 (13.52%) cases. Incidence rate (density) of: VAP/1000 MV- days, UTI /1000 UC- days and CLA-BSI/1000 CL- days were 11,15 ± 2.5, 6.82 ± 0.81, 2.35 ± 1.54.respectivelly. *P.aeruginosa* was the pathogen most frequently responsible for VAP 69/108 (63.88%). Mean frequency of VAP, UTI and CLA-BSI with *P.aeruginosa* etiology was 69/493 (14.28%), 32/299 (11.1%) and 7/127 (5.77%) respectively. The mean density of *P.aeruginosa* infection amounted to 2.43/1000 patient-days. The decrease was observed in the total number of DA-HAIs caused by the *P.aeruginosa* from 15.75% and 3.23/1000 patient-days in 2011 to 5.0% and 1.17/1000 in 2016 (*p* = 0.0104, *p* = 0.0348). Starting from 2016 to 2019 incidence and density of *P.aeruginosa* DA-HAIs increased to 12.33% and 2.63/1000 (*p* = 0.1388, *p* = 0.0818). *P.aeruginosa* was susceptible to ceftazidime, cefepime, amikacin, meropenem, ciprofloxacin, colistin, in 55.55, 58.33, 70.37, 53.73, 50, and 100% respectively. MDR characterised it in 40% in 2011 and 66.7% in 2019, (*p* = 0.177).

**Conclusions:**

The study revealed a changeable prevalence of *P. aeruginosa* strain infections; however their frequency was never highest in our ICU patients as it presented in the last years in Europe. The study showed a significant decrease in 2016 and increase in 2019, a nearly 3-fold increase of *P.aeruginosa* infections among Gram-negative strain infections, and a 2-fold increase of the *P.aeruginosa* DA-HAIs frequency between 2016 and 2019 as well as an increased resistance. Microbiological analysis of DA-HAIs in each hospital should be a standard method used in hospital infection control and antibiotic policy. In the case of *P.aeruginosa,* in order to minimize transmission, preventive infection methods should be assessed mainly in case of VAP.

## Background

*Pseudomonas aeruginosa* is a Gram-negative bacillus which belongs to the *Pseudomonadacae* family [1]. In hospital environments, it can colonize wet places including medical ventilators, oxygen respirators, humidifiers, sinks, taps, toilets and dialysis machines [2]. Risk factors associated with *P.aeruginosa* infections are: chronic obstructive pulmonary disease, diabetes, cystic fibrosis, immunosuppression (after the organ or bone marrow transplantation), severe kidney and liver failure, and multi-organ injury [2]. *P.aeruginosa* infections are the most common cases treated in intensive care units (ICU) including hematological, surgical and burn units. Clinical forms of *P.aeruginosa* infections are hospital acquired pneumonia (HAP) including ventilator associated pneumonia (VAP), urinary tract infection (UTI), bloodstream infections (BSI), including central line associated bloodstream infections (CLA-BSI), burn wound infections, skin and soft tissue infections, surgical site infections, decubitus ulcers, ocular infections, central nervous system infection, bone and joint infections and finally otitis interna [3]. Published results from the European Centre for Disease Prevention and Control (ECDC) Register (2016) indicate that *P.aeruginosa* infections in hospital environments of Polish and European ICUs are responsible for 20.8 and 15.9% VAP, 14.7 and 10.5% UTI, 11.1 and 27.8% CLA-BSI respectively [4]. Global mortality of patients with *P.aeruginosa* infection is about 20% and is higher in cases of VAP (30%) and bacteremia (50%) [5]. Polish data from EARS-Net (2017) indicate a high sensitivity of the *P.aeruginosa* strains to carbapenems (76.8%), aminoglycosides (74.5%), ceftazidime (75.4%) and multidrug resistance (MDR) which was verified in 22.8% of the study cases [6]. In the same study, the incidence of *P.aeruginosa* MDR strains in European Union countries was 30.8% [6]. According to a recent multicenter registry of device associated-healthcare-associated infections (DA-HAIs) records, *P.aeruginosa* plays a critical role as an etiological factor of VAP in Europe, which indicates the importance of the problem [7]. The study aimed to analyze ICU acquired DA-HAIs and their microbial factors (Gram-positive, Gram-negative bacteria and fungi) in terms of the incidence of *P.aeruginosa* infections and its susceptibility within 8.5-years of observation.

## Methods

Four thousand ten patients hospitalised in the 20-bed Intensive Care Unit of the University Hospital in Wroclaw from 01.01.2011 to 31.06.2019 were included in the study. The Bioethics Committee of Wroclaw Medical University gave its approval of this study (KB-579/2016). The patients’ written consent was not required by the Ethics Committee of Wroclaw Medical University, because a statement covering patients’ data confidentiality was fully respected during data collection and the manuscript preparation.

The study used the data on ICU acquired pathogens of DA- HAIs. The data concerning clinical forms of the infection and microbiological data related to all *P.aeruginosa* strains susceptibility were collected prospectively and obtained from DA-HAIs monthly reports, hospital infection registration cards and the electronic hospital databases from Microbiological Laboratory. The study included a patients with DA-HAIs *P.aeruginosa* only once, as well as only one isolate responsible for infection from a single patient, were included. The same methodology was used for another pathogens collection. The study did not analyse *P.aeruginosa* strains responsible for colonisations. Data collected prospectively for epidemiological purposes concerning patient-days of hospitalization, and ventilator days, urinary catheter days, central line days were also included in the study.

DA-HAIs were diagnosed according to definitions adopted by ECDC in patients hospitalized for more than 48 h [8]. VAP was defined as PNEU 1–3 and diagnosed on a basis new or progressive or persistent infiltrate with consolidation or cavitation in chest radiographs, fever > 38 C, leukocytosis ≥12,000 WBC/ mm^3^ or leukopenia < 4000 WBC/mm^3^ and purulent sputum, cougth, dyspnea, tachypnea, rales, rhonchi, wheezing, worsening gas exchange and positive microbiological examinations/results from tracheal aspirate or blood. Microbiologically confirmed symptomatic urinary tract infection at the patient with an involved urinary catheter (UC) was diagnosed based on fever >38C, localised pain at the involved site or suprapubic tenderness, urgency, frequency, dysuria, pyuria and positive urine culture. Central Line-Associated Bloodstream Infection was defined as CRI 3 (microbiologically confirmed central venous catheter (CVC) related bloodstream infection) and diagnosed on a basis clinical symptoms as fever >38C or hypothermia < 36 C, hypotension, chills and positive microbiological laboratory results from the catheter tip and from the blood which was not related to an infection at another site [8, 9]. The supervision process of hospital infections was carried out routinely by the physician, microbiologist and two nurses (a departmental infection control team) and the Hospital Committee for Infection Control.

Infections were diagnosed microbiologically in the certified Microbiological Laboratory of the University Hospital in Wroclaw. Mini-bronchoalveolar lavage (mini-BAL) or BAL with > 10^4^ colony forming unit (CFU) / mL were used for microbiological diagnosis of ventilation associated pneumonia (VAP). In case of bacterial loads in urine > 10^3^ or < 10^5^ CFU/mL (no more than 2 pathogens) and pyuria, urinary tract infection was diagnosed [8, 9]. CLA-BSI was recognised in the case of positive culture from the blood and from the tip of the central venous catheter (> 15 CFU in a semi-quantitative method or > 10^3^ CFU/mL in a quantitative method) [8, 9]. An automated method (Gram-negative (GN) and Gram-positive (GP) card) in the Vitek 2 Compact (bioMerieux, Paris, France) was carried out for identification of Gram-positive and Gram-negative bacteria [10]. Then, all of the strains were tested manual methods using specific biochemical tests. Susceptibility of microorganisms, as well as the interpretation of the results, was determined with the use of the disc diffusion method on Muller Hinton (BioRad, Berkley, CA, USA) substrate and by using strips with antibiotic concentration gradient E-test (bioMerieux, Paris, France) for MIC (minimum inhibitory concentration) assessment. Antimicrobial susceptibility assessment to colistin was performed using Broth Microdilution Method (BMD). Starting from 2017, for microbiological diagnosis of infections molecular multiplex PCR (Polymerase Chain Reaction) methods (FILMARRAY Respiratory Panel and Blood Culture Identification Panel, BioFire Diagnostics, Salt Lake City, USA) were also used. All diagnostic methods were made according to the protocol adopted by and consistent with the European Committee on Antimicrobial Susceptibility Testing (EUCAST) methodology as they are the only one suitable criteria in our country [10]. Multidrug resistance of the *P.aeruginosa* strain was defined as non-susceptible to ≥1 agent in ≥3 antimicrobial categories [11].

Incidence of *P.aeruginosa* DA-HAIs was calculated using the equation: the number of *P.aeruginosa* infections / the number of patient-days in the given time × 1000. Incidence rate (density) of DA-HAIs were calculated using the equation: the number of DA-HAIs /1000 patient-days, whereas incidence rate (density) of: VAP, UTI, CLA-BSI dividing the number of infections /1000 device utilisation days (mechanical ventilator (MV)- days, urinary catheter (UC)- days, central line (CL) -days).

STATISTICA program version 13.1 (StatSoft Inc., Tolusa, USA) was used for statistical analyses. Descriptive statistics were performed for all study variables. Discrete variables are expressed as counts (percentage) or mean and SD (standard deviation). Distribution of qualitative variables was analysed using Chi-square test, Chi-square test with Yates correction and Fishers’s exact tests which were used adequately to the strength of the group. *p* value < 0.05 was considered as statistically significant.

## Results

**Fig. 1:**
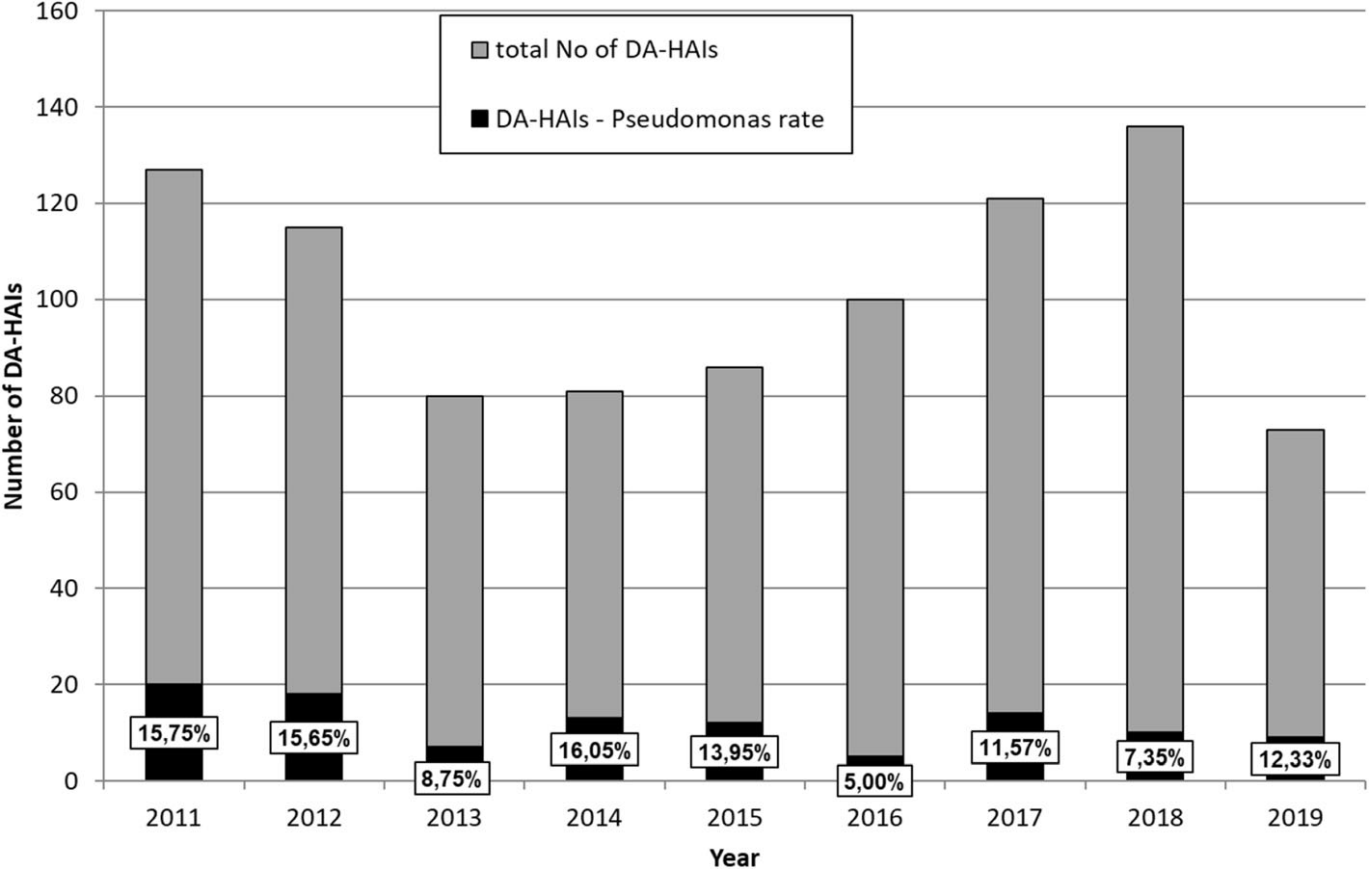
Percentage of *Pseudomonas aeruginosa infections* (DA-HAIs-Pseudomonas) among the total number of DA-HAIs. Abbreviation: DA-HAIs, Device Associated –Healthcare Associated Infections

Four thousand ten patients (1583 females and 2427 males; average age 60.9 ± 17) treated in the ICU during 44,154 patient-days of hospitalization were included in the study. The DA-HAIs (*n* = 799) was diagnosed in 19.9% of all the hospitalised patients. The total number of 919 bacterial and fungal strains were diagnosed at patients with DA- HAIs. Incidence rate (density) (mean ± SD) of DA-HAIs was 17,62 ± 1,98/1000 patient-days, whereas incidence rate (density) of: VAP/1000 MV- days, UTI /1000 UC- days and CLA-BSI/1000 CL- days were 11,15 ± 2.5, 6.82 ± 0.81, 2.35 ± 1.54.respectivelly. The clinical forms of DA-HAIs has frequency: VAP 407/799 (50.9%), UTI 299/799 (37.4%), CLA-BSI 120/799 (15.01%) and were caused by 493/919 (53.64%), 299/919 (32.53%), 127/919 (13.82%) strains (bacterial and fungal) respectively. Among this strains (*n* = 919) the Gram-negative microorganisms (*n* = 656) were dominant and they constituted 656 /919 (71.38%), while Gram-positive bacteria and fungi constituted 198 /919 (21.54%) and 65/919 (7.07%), respectively. *P.aeruginosa* strains (*n* = 108) were responsible for 108/799 (13.52%) of DA-HAIs and constituted 108/656 (16.46%) of the total Gram-negative bacilli. The percentage of *P.aeruginosa* infections among Gram-negative bacterial infections was 20/79 (25.32%), 5/76 (6.58%) and 9/50 (18%) in 2011, 2016 and 2019 respectively. Analysis of DA-HAIs pathogens and the incidence of *P.aruginosa* infections in the context of Gram-negative bacteria infections are presented in Table 1. *P.aeruginosa* was the pathogen most frequently responsible for VAP 69/108 (63.88%), then UTI 32/108 (29.63%) and CLA-BSI 7/108 (6.48%). Mean frequency of VAP, UTI and CLA-BSI with *P.aeruginosa* etiology was 69/493 (14.28%), 32/299 (11.1%) and 7/127 (5.77%) respectively. The incidence of *P.aeruginosa* infections was changeable. A decrease was noted in the total number of hospital infections caused by the *P.aeruginosa* strain from 20/127 (15.75%) in 2011 to 5/100 (5.0%) in 2016, *p* = 0.0104. As presented in Fig. 1, starting from 2016 to 2019, the incidence of *P.aeruginosa* DA-HAIs increased to 9/57 (12.33%), *p* = 0.1388. The mean incidence of *P.aeruginosa* infection corresponded to 2.43/1000 patient-days. A decrease was noted in the total number of DA-HAIs caused by *P.aeruginosa* strain from 3.23/1000 patient-days in 2011 to 1.715/1000 in 2016, *p* = 0.0348. Starting from 2016 to 2019 (one half of a year) the density of *P.aeruginosa* DA- HAIs increased to 2.63/1000, *p* = 0.1388.

**Table 1:** Etiological analysis of Device Associated –Healthcare Associated Infections and the percentage of *Pseudomonas aeruginosa* infections among gram-negative bacterial infections

	Year									***p***-value
	2011	2012	2013	2014	2015	2016	2017	2018	2019 (01–06)	
G(−), n (%)	79 (62,2)	82 (71,3)	57 (71,25)	68 (83,9)	68 (79,1)	76 (76)	85 (70,25)	91 (66,9)	50 (68.5)	
*Pseudomonas aeruginosa*, n (%)	20 (25.32)	18 (21.95)	7 (12.28)	13 (19.12)	12 (17.64)	5 (6.58)	14 (16.47)	10 (10.99)	9 (18)	*p* = 0.334 *p = 0.016 **p = 0.0467
G(+), n (%)	39 (30.7)	31 (27)	10 (12.5)	9 (11.1)	13 (15.1)	19 (19.0)	30 (24.7)	30 (22.06)	17(23.3)	
Fungi, n (%)	9 (7.1)	2 (1.75)	13 (16.25)	4 (4.9)	5 (5.8)	5 (5.0)	6 (5.0)	15 (11.03)	6 (8.2)	

Notes: Data are presented as number and percentage value. *p* value was calculated for the years 2011 vs. 2019; * *p* value for the years 2011 vs 2016; ** *p* value for the years 2016 vs 2019; *Abbreviations*: *G(−)* Gram negative, *G(+)* Gram positive, n number

Detailed analysis of the incidence of *P.aeruginosa* DA-HAIs is shown in Table 2.

**Table 2:** Incidence of *Pseudomonas aeruginosa* DA-HAIs per 1000 patient-days

	Year									Sum or mean	***p***-value
	2011	2012	2013	2014	2015	2016	2017	2018	2019 (01–06)		
No. of DA-HAIs	20	18	7	13	12	5	14	10	9	108	
No. of patient-days	6190	5327	4445	4234	4143	4251	6306	5829	3427	44,152	
No. of DA-HAIs/1000 patient-days	3.23	3.38	1.57	3.07	2.896	1.176	2.22	1.715	2.63	2.431	*p* = 0.6044 *p = 0.0348 **p = 0.1388

Notes: *p* was calculated for the correlation of data 2011 vs. 2019; * *p* value for 2011 vs. 2016; ** *p* value for 2016 vs 2019

*Abbreviations*: *HAIs* Health care associated infections, *No* Number

The contribution of *P.aeruginosa* strain in the individual clinical forms of DA-HAIs (VAP, UTI, CLA-BSI) is presented in Table 3.

**Table 3:** The contribution of *P.aeruginosa* strain in the individual clinical forms of DA-HAIs (VAP, UTI, CLA-BSI). Data are presented as numbers

Year	2011	2012	2013	2014	2015	2016	2017	2018	2019	2011–2019
**No of VAP**	67	57	35	43	56	56	70	74	35	493
**No of VAP-Pseudomonas**	11	12	5	7	7	4	11	6	6	69
**No of UTI**	41	34	29	28	24	34	42	42	25	299
**No of UTI Pseudomonas**	5	5	2	5	4	1	3	4	3	32
**No of CLA-BSI**	19	24	16	10	6	10	9	20	13	127
**No of CLA-BSI Pseudomonas**	4	1	0	1	1	0	0	0	0	7

Analysis of the susceptibility of *P. aeruginosa* strain related to piperacylin/tazobactam, ceftazidime, cefepime, imipenem, meropenem, amikacin, gentamicin, ciprofloxacin, and colistin is presented in Table 4. During the study period, an increase in the resistance was found in the case of the majority of previously enumerated antibiotics. It was also found that all of the *P.aeruginosa* strains were susceptible to colistin.

**Table 4:** Percentage of the susceptibility of *Pseudomonas aeruginosa* strain to selected antibiotics .Data are presented as number and (%) of the susceptible strains in selected years

	Year									***p***-value
Antibiotics/Strains (n)	2011	2012	2013	2014	2015	2016	2017	2018	2019 (01–06)	
	20	18	7	13	12	5	14	10	9	–
Piperacillin/ tazobactam	8 (40)	9 (50)	3 (42.9)	7 (53.8)	6 (50)	3 (60)	13 (92.9)	5 (50)	2 (22.2)	*p* = 0.311 *p = 0.001
Ceftazidime	10 (50)	7 (38.9)	3 (42.9)	8 (61.5)	7 (58.3)	3 (60)	13 (92.9)	6 (60)	3 (33.3)	*p* = 0.335 *p = 0.0049
Cefepime	13 (65)	9 (50)	3 (42.9)	5 (38.5)	7 (58.3)	3 (60)	14 (100)	6 (60)	3 (33.3)	*p* = 0.225 *p = 0.0008
Imipenem	11 (55)	7 (38.9)	2 (28.6)	7 (53.8)	7 (58.3)	1 (20)	11 (78.6)	7 (70)	2 (22.2)	*p* = 0.129 *p = 0.0335
Meropenem	11 (55)	9 (50)	2 (28.6)	7 (53.5)	8 (66.7)	1 (20)	11 (78.6)	6 (60)	2 (22.2)	*p* = 0.1296 *p = 0.0335
Amikacin	11 (55)	13(2.7)	6 (85.7)	8 (61.5)	10 (83.3)	4 (80)	14 (100)	7 (70)	3 (33.3)	*p* = 0.427 *p = 0.008
Gentamicin	11 (55)	9 (50)	5 (71.4)	4 (30.8)	8 (66.7)	2 (40)	14 (100)	7 (70)	3 (33.3)	*p* = 0.427 *p = 0.0008
Ciprofloxacin	9 (45)	6 (33.3)	4 (57.1)	4 (30.8)	7 (58.3)	2 (40)	14 (100)	6 (60)	3 (33.3)	*p* = 0.4118 *p = 0.0008
Colistin	8 (100)	10 (100)	3 (100)	4 (100)	5 (100)	2 (100)	–	3 (100)	9 (100)	

Notes: *p* was calculated for the correlation of data 2011 vs. 2019; * *p* value for 2017 vs. 2019 *Abbreviation*: *n* number of *P.aeruginosa* strains

In 2011, 8/20 (40%) of the *P.aeruginosa* strains were MDR, whereas in 2019 it was 6/9 (66.7%), *p* = 0.1771. The total number of the MDR *P.aeruginosa* strains was doubled between 2018 and 2019: 3/10 (30%) vs 6/9 (66.7%), *p* = 0.1789 and significantly increased between 2017 vs 2019: 0/14 (0%) vs 6/9 (66.7%), *p* = 0.0008. During this study, multidrug resistance was found in (41/108) 37.96% of the *P.aeruginosa* strains.

## Discussion

Our study showed that despite the changeable incidence of the *P.aeruginosa* infection and its increased resistance during the study period, starting from 2016, the *P.aeruginosa* has constituted a critical epidemiological problem. Nevertheless, this “alert pathogen” never was the most common in Gram negative stains responsible for DA-HAIs. On the other site, analysis of clinical form of DA-HAIs showed significant involvement *P.aeruginosa* in VAP epidemiology. From a clinical point of view, critical information found in this study, was significant increase of *P.aeruginosa* MDR strains, which obligate physicians to use colistin in the empirical treatment of late-VAP in our centre. Interestingly, oscillation in *P.aeruginosa* infections rate and its decreased frequency starting from 2011 (to 2016) was in opposition to increased frequency of *A. baumannii* DA-HAIs in the same ICU [12]. The average incidence of *P.aeruginosa* infections in our study (13.58%) was higher than in the ICUs of other Polish University Hospitals (6.38%) but lower than in another Polish non-university ICU (16.9%) [13, 14]. In comparison to our study, a 10-year observational study (2007–2016) in a district Hospital in Southern Poland showed that *P.aeruginosa* infections in ICU patients were observed less frequently (8%) [15]. Similarly, in a multi-centre study conducted in a non-teaching hospital in Poland (2013–2015), *P.aeruginosa* VAP was diagnosed less frequently (6%) in comparison to our study [16]. Published analysis of microbiological pathogens responsible for VAP (2012–2014) at our ICU showed that the most common VAP pathogen was *Acinetobacter baumannii*, whereas *P.aeruginosa* infections were diagnosed in 17% of VAP [17]. These findings showed a higher number of *P.aeruginosa* infections in comparison to the results of our study [17]. The mean frequency of *P.aeruginosa* infections (VAP, UTI) in our study was nearly similar to the frequency of *P.aeruginosa* infections in Polish hospitals that participated in the ECDC multi-center report in 2016 (VAP 15.9%; UTI 10.5%), but was definitely lower in the case of CLA-BSI (27.8%) [4]. Interestingly, *P.aeruginosa* infections including VAP, UTI and CLA-BSI in our study were lower in comparison to the mean frequency of *P.aeruginosa* infections in Europe (VAP 20.8%, UTI 14.7%, CLA-BSI 11.1%) in the same report [4]. Our results contradict the ECDC report of 2017 (without data from Poland) in which *P.aeruginosa* was the most common pathogen responsible for pneumonia in Europe and constituted 19.9% infections (33.3% in Slovakia, 32.4% in Hungary, 29.2% in Portugal, 24% in Spain, 23.1% in France, 19.4% in Italy, 17.1% in Belgium, 16.1% in Germany and 7.2% in the United Kingdom) [7]. The incidence of DA-HAIs with *P.aeruginosa* etiology in our study was lower in comparison to other Polish reports (one centre studies), where *P.aeruginosa* caused pneumonia in 18%, UTI in 30% and blood infections in 10.1% of the patients [14]. Incidence of VAP with *P.aeruginosa* etiology nearly three-fold higher than our results was demonstrated in the Brazilian study (39.5%) [18]. Also, the incidence of *P.aeruginosa* VAP (23,2%), UTI (14,9%), CLA-BSI (8,3%) in the Greek study was higher in comparison to our findings [19].

In this study, similarly to our earlier published data from 2011 to 2016, the Gram-negative pathogens were predominant and *A.baumannii* was still the most common, particularly in VAP (data not shown) [12]. A lower incidence of *P.aeruginosa* among Gram-negative strains responsible for VAP was found in a single-day multicenter study (*n* = 205) conducted in the Mazovian region in Poland (PPIC–Polish Prevalence of Infection in Intensive Care) (2014) [20]. *P.aeruginosa* was responsible for 4.1% of the infections and constituted 14.9% of the Gram-negative strains responsible for pneumonia [20]. Similar to our results, the Gram-negative strains were the most common cases of ICU infections in several large studies that were published, indicating where the incidence of *P.aeruginosa* infections was higher in comparison to our study [21–24]. In a single-day, multicenter study (*n* = 10,038), European Prevalence of Infection in Intensive Care (EPIC) (1995), it was demonstrated that the most common pathogens of infections in the ICUs were Enterobacteriaceae (34.4%), while *P.aeruginosa* was responsible for 28.7% of all infections (*n* = 4501, 21]. Another multicenter European study revealed that the incidence of *P.aeruginosa* infection in patients with sepsis (*n* = 3147) was 14%. In comparison, the Extended Study on Prevalence of Infection in Intensive Care (EPIC II) /(EPICIII) reported 19.9%/23% [22–24]. In a worldwide sepsis study ICON (Intensive Care over Nations) (2012), the prevalence of *P.aeruginosa* infections in ICU patients (*n* = 10,069) was 16.3% [25]. In the first Polish multicenter study (*n* = 1043) from 2004, the most common pathogens that caused sepsis were Gram-negative (48%); where *P.aeruginosa* consisted 14.2%, nearly equal to our study, Gram-positive (43%), and fungi (21%) [26]. Moreover, there are published reports demonstrating a large variation in the incidence of *P.aeruginosa* infections in various geographic regions (12.9% in North America, 14.8% in Africa, 17.1% in Western Europe, up to 28.7% in Asia and 28.9% in Eastern Europe) [23].

The results of this study showed a high level of multidrug resistance of the *P.aeruginosa* strain (37.96%), which is significantly higher than the mean percentage (22.8%) of Pseudomonas MDR in Poland (based on data of European Antimicrobial Resistance Surveillance Network, EARS-Net 2017) [6]. Additionally, the percentage (66.7%) of *P.aeruginosa* MDR strain infections in our study in 2019 was significantly higher than in 2017 in France (10.6%), Germany (7.2%), Belgium (6.6%), Sweden (3.1%), Spain (10.9%), Greece (32.4%), Italy (17.5%) and in 2014 in USA (16.7–21.7%) [6, 27]. Analysis of EARS-Net data from Poland (2014–2017) shows changeable, but lower than in our study, frequency of *P.aeruginosa* MDR which was 26.7, 29.6%, 20.6.%, 22.8 respectively in 2014, 2015, 2016, and 2017 [6]. Interestingly, in this study (data from 2018) the 60% susceptibility of *P.aeruginosa* strain to fluoroquinolones is lower than in Germany (85.8%), France (84.9%), Spain (79.9%), Italy (74.9%) and relatively close to those found in Poland (62.8%) and USA (69.8%) [6, 27]. Also, the percentage of susceptible strains to aminoglycosides (70%) in our study is lower than in Germany (95.2%), France (89.1%), Spain (87.5%), Italy (82%), USA (82.8%) and nearly the same as in Greece (69.8%) [6, 27]. Additionally, susceptibility to carbapenems (60–70%) in our study is also lower to susceptibility occurring in Germany (87.3%), Italy (80,1%), Spain (81.6%), Poland (75.8%) and similar to USA (74.2%) and Greece (60.7%) [6, 27]. Two reports coordinated by INICC and covering 50 and 43 developing countries, have shown lower resistance of the *P.aeruginosa* strain to imipenem (33.7–43.2%) or meropenem (39.4–43.48%) when compared to our results [28, 29]. Nonetheless, the study has numerous limitations. Firstly, it is a single-centre study (with possibility of biases), so an influence on both the incidence and susceptibility of *P. aeruginosa* strains in Poland, can be different. Secondly, the analysis was carried out on the basis of ICU acquired DA-HAIs only, so it could be the reason of a high percentage of *P.aeruginosa* MDR strains. Thirdly, the results of study may have been affected by the heterogeneity of the patient group, patients conditions, severity of the disease, used methods for prevention of DA-HAIs and for the spreading of MDR strains. Fourthly, despite DA-HAIs preventive bundles (including VAP, UTI, CLA-BSI preventive bundles and compliance with particular components of this bundles) and hand hygiene compliance were monitored periodically at our ICU [30], we did not presented them because it was not the aim of this study. Nevertheless according to published data, it was shown that DA-HAIs preventive bundles are associated with DA-HAIs reduction (also length of hospital stay and hospital cost) [30–32]. Also, the implementation of Hand Hygiene programs contributed to DA-HAIs reduction and mortality [33]. Finally, we did not find any published studies showing such long period *P.aeruginosa* infections analysis as well as showing an incidence of infection with the *P.aeruginosa* concerning the patient-days of hospitalization, which did not allow us to carry out a comparative analysis.

## Conclusions

The study revealed a changeable prevalence of *P. aeruginosa* infections but their frequency was never most common in ICU patients as was found in the last years in Europe. The incidence of *P.aeruginosa* strain infections in ICU patients had a significant increase in 2019, a nearly three-fold increase of the percentage of *P.aeruginosa* among Gram-negative strains, and a two-fold increase of the frequency of *P.aeruginosa* HAIs between 2016 and 2019. Furthermore, a significant increase of multi-resistance and resistance to anti-pseudomonal penicillin, cephalosporins III/IV generation, fluoroquinolone, carbapenems were found. The implementation of infection-control measures aiming at the inhibition of *P.aeruginosa* strains transmission, as well as preventing infections, especially VAP and multi-resistance, is necessary to tackle this problem at our ICU. Microbiological analysis of DA-HAIs in each hospital should be a standard method used in hospital infection control and antibiotic policy.

## Data Availability

The datasets collected and analysed during this study are available and can be accessed from Agnieszka Litwin (e-mail: agalee9@op.pl)

## Abbreviations

BAL: Bronchoalveolar lavage
CFU: Colony forming unit
CL: Central line
CLA-BSI: Central line-associated bloodstream infection
CRI: Catheter related infection
CVC: Central venous catheter
DA-HAIs: Device associated - healthcare associated infections
EARS-Net: European Antimicrobial Resistance Surveillance Network
ECDC: European Centre for Disease Prevention and Control
EPIC: European Prevalence of Infection in Intensive Care
EUCAST: European Committee on Antimicrobial Susceptibility Testing
GN: Gram Negative
GP: Gram Positive
HAP: Hospital acquired pneumonia
ICU: Intensive Care Unit
ICON: Intensive care over nations
INICC: International Nosocomial Infection Control Consortium
MDR: Multidrug-resistant
MV: Mechanical ventilator
PCR: Polymerase chain reaction
PNEU: Pneumonia
SD: Standard deviation
USA: The United States of America
UC: Urinary catheter
UTI: Urinary tract infection
WBC: White blood cells
VAP: Ventilator-associated pneumonia
